# Investigating the dynamic relationship between joint function outcomes and kinesiophobia following total knee arthroplasty

**DOI:** 10.3389/fpsyg.2026.1765989

**Published:** 2026-02-24

**Authors:** Caijin Wen, Peiyao Wu, Qin Qin, Xi Luo, Lu Wei, Jing Zhang

**Affiliations:** 1 School of Nursing, North Sichuan Medical College, Nanchong, Sichuan, China; 2 Nursing Department of the Affiliated Hospital of Panzhihua University, Panzhihua, Sichuan, China; 3 Orthopedics Department of Panzhihua Central Hospital, Panzhihua, Sichuan, China

**Keywords:** cross-lagged model, joint function, kinesiophobia, longitudinal study, total knee arthroplasty

## Abstract

**Objective:**

To investigate the temporal trends and bidirectional predictive relationships between joint function outcomes and kinesiophobia in patients following total knee arthroplasty (TKA).

**Methods:**

Using a convenience sampling method, 242 patients who underwent TKA in the orthopedics departments of two Grade A Tertiary hospitals in Panzhihua City between October 2024 and March 2025 were selected as study participants. The Western Ontario and McMaster Universities Osteoarthritis Index (WOMAC) and the Tampa Scale for Kinesiophobia-11 (TSK-11) were used to assess joint function and kinesiophobia at 1 month (T1), 3 months (T2), and 6 months (T3) postoperatively. A cross-lagged analysis was employed to analyze the causal relationships between the variables.

**Results:**

Joint function in TKA patients improved significantly over time, while kinesiophobia levels gradually decreased, with statistically significant differences in scores across all time points (*P* < 0.05). By 6 months post-surgery, the excellent and good outcome rate reached 72.31%, indicating that joint function still retained potential for further improvement. Cross-lagged analysis revealed a bidirectional causal relationship between joint function and kinesiophobia. In the early rehabilitation phase (T1–T2), kinesiophobia was a stronger predictor of joint function (β = 0.368, *P* < 0.001). Conversely, in the middle to late rehabilitation phase (T2–T3), joint function became a more prominent predictor of kinesiophobia (β = 0.218, *P* < 0.001).

**Conclusion:**

We found a bidirectional relationship between joint function and kinesiophobia during TKA recovery, with the dominant influence shifting from kinesiophobia early on to joint function later. This suggests that rehabilitation strategies should be phased, initially addressing fear and later focusing on functional training to optimize outcomes.

## 1 Introduction

Total knee arthroplasty (TKA) is an established and effective surgical procedure for end-stage knee osteoarthritis with severe pain and dysfunction that is refractory to conservative management. It demonstrates significant efficacy in correcting limb alignment, alleviating pain, and improving patients' functional capacity for daily activities (Dong et al., 2023). The increasing adoption of TKA in recent years reflects a convergence of demographic, technological, and sociocultural shifts, including global population aging, continuous advancements in medical science, and changing health expectations among patients. This has led to a consistent rise in procedural volumes worldwide. For instance, approximately 100,000 TKAs are performed annually in the United Kingdom (National Joint Registry 14th Annual Report 2017, 2017), with the figure surpassing 700,000 in the United States (Bavaria et al., 2019). Projections indicate that by 2030, the annual incidence of primary TKA in the United States will reach 3.48 million, representing a 673% increase from 2005 levels (Kurtz et al., 2017). A similar upward trajectory is evident in other countries (NiemeläInen et al., 2017; Tang et al., 2016). Although clinical data indicate favorable long-term prosthesis survival rates and radiological outcomes for TKA (Lange et al., 2018), a non-negligible proportion of patients report suboptimal recovery after TKA. Specifically, 14%−53% experience persistent or intermittent knee pain, 7%−50% exhibit inadequate functional recovery (Atkinson, 2017), and 15%−30% express dissatisfaction with the overall surgical outcome (DeFrance and Scuderi, 2023). Therefore, an in-depth investigation into the key factors and underlying mechanisms influencing postoperative joint functional recovery is of considerable clinical importance for improving the quality of patient rehabilitation. Kinesiophobia, also known as fear of movement, refers to an excessive fear of pain or re-injury that leads to activity avoidance (Reneman et al., 2003) and is a critical psychological factor affecting joint functional recovery (Yang et al., 2024). Reported incidence of kinesiophobia following TKA ranges from 30% to 60% (Morri et al., 2020; He et al., 2023). The Fear-Avoidance Model posits that persistent pain and dysfunction are closely linked to patients' fear of pain and the resulting activity avoidance, often forming a vicious cycle where these factors mutually reinforce each other (George et al., 2003). Multiple cross-sectional studies, both domestic and international, have demonstrated a significant negative association between kinesiophobia and joint functional outcomes. High levels of kinesiophobia can substantially reduce patients' engagement in rehabilitation activities, thereby delaying the functional recovery process (Fan et al., 2025; Thoma et al., 2021; Alshahrani et al., 2022). Conversely, successful experiences of functional improvement may, to some extent, alleviate kinesiophobia by enhancing patients' self-efficacy (Candiri et al., 2023). However, cross-sectional study designs cannot elucidate temporal sequences or causal relationships between variables. Therefore, this study employs a longitudinal design combined with Cross-Lagged Model (CLM) analysis to systematically explore the temporal interaction patterns between joint function and kinesiophobia in patients following TKA and to attempt to reveal the potential dominant directionality and interactive mechanisms between these two constructs. This study aims to guide the development of timely and personalized intervention strategies that address both psychological and functional recovery after TKA.

## 2 Materials and methods

### 2.1 Participants

This study employed a convenience sampling method to recruit patients who underwent TKA in the orthopedic departments of two tertiary Grade-A general hospitals in Panzhihua City between October 2024 and March 2025. Inclusion criteria were as follows: Met the diagnostic criteria for primary knee osteoarthritis and was indicated for TKA; Scheduled for unilateral, primary total knee arthroplasty; Possessed normal comprehension and communication abilities; Provided informed consent and voluntarily participated in the study; Underwent surgery using a cemented fixation technique and completed with conventional surgical instruments. Exclusion Criteria: History of psychiatric disorders, cognitive impairment, or severe hearing impairment affecting communication and scale assessment; Presence of other conditions explicitly affecting sensory or motor function of the lower limbs, including but not limited to uncontrolled diabetic peripheral neuropathy, lumbar spinal stenosis with radicular symptoms, lower-limb motor impairment after stroke, Parkinson's disease, severe lower-limb arterial occlusive disease, and prior history of spinal or peripheral nerve injury.

The required sample size was estimated using a Monte Carlo simulation (Thoemmes et al., 2010). Based on a prior study reporting a correlation coefficient of 0.49 between joint function and kinesiophobia (Alshahrani et al., 2022), the autoregressive and cross-lagged path coefficients in the proposed cross-lagged model were both set to 0.5 for power analysis. The simulation results indicated that a sample size of 130 participants would yield statistical power exceeding 0.95 (0.954–0.981) for all path coefficients. Accounting for an estimated 20% attrition rate, a final minimum sample size of 163 participants was determined. The study protocol was approved by the Hospital Ethics Committee (Approval No. 2024-10-005).

### 2.2 Measurement instruments

#### 2.2.1 General information questionnaire

A General Information Questionnaire was developed by the research team based on a comprehensive review of relevant literature. The questionnaire consisted of two main sections: Demographic Data: including gender, age, Body Mass Index (BMI), marital status, educational level, place of residence, and monthly household income; Disease-Related Data: including preoperative joint function scores, Kellgren-Lawrence grading, comorbidities, and duration of illness.

#### 2.2.2 Western Ontario and McMaster Universities Osteoarthritis Index (WOMAC)

Postoperative knee joint functional outcomes were assessed using the Western Ontario and McMaster Universities Osteoarthritis Index (WOMAC). This scale was originally developed by Bellamy et al. (1988) and subsequently translated and cross-culturally adapted into Chinese by Xie et al. (2008). The scale comprises three domains: pain (5 items), stiffness (2 items), and physical function (17 items), totaling 24 items. A 5-point Likert scale (0–4) is used for scoring, with a total possible score ranging from 0 to 96. Higher total scores indicate more severe osteoarthritis symptoms. Total scores are interpreted as follows: < 21 indicates mild severity, 21–48 indicates moderate severity, and >48 indicates severe severity. A postoperative WOMAC score of < 48 served as the predefined cut-off for “good-to-excellent” functional outcome (Copsey et al., 2019). Treatment efficacy was further classified using the therapeutic index formula: [(preoperative score–postoperative score) / preoperative score] × 100%. The resulting percentages were graded as: excellent (>75%), good (50%−75%), fair (25%−49%), or poor (< 25%) (Zheng, 2002). In the original scale, the intra-class correlation coefficients for the pain, stiffness, physical function domains, and the total score were 0.937, 0.914, 0.856, and 0.921, respectively, demonstrating good reliability and validity. In the present study, the Cronbach's α coefficients for the total scale across the three measurement time points were 0.882, 0.888, and 0.887, respectively.

#### 2.2.3 Tampa Scale for Kinesiophobia-11 (TSK-11)

The level of kinesiophobia was assessed using the Chinese version of the Tampa Scale for Kinesiophobia-11 (TSK-11). This scale was originally revised by Woby et al. (2005) and subsequently translated and cross-culturally adapted into Chinese by Cai et al. (2019). It consists of 11 items, which can be evaluated across three dimensions: activity cognition (6 items), activity behavior (3 items), and activity attitude (2 items). The scale employs a 4 - point Likert rating system, ranging from “strongly disagree” (1 point) to “strongly agree” (4 points). The total score ranges from 11 to 44, with higher scores indicating a greater severity of kinesiophobia. The Chinese version of the TSK-11 has demonstrated an internal consistency reliability (Cronbach's α) of 0.883 and a test-retest reliability of 0.798. In the present longitudinal study, the overall Cronbach's α coefficients for the scale across the three measurement time points were 0.931, 0.920, and 0.921, respectively.

### 2.3 Data collection

The follow-up time points for this study were selected based on evidence synthesis (Zhou et al., 2019) and expert consensus (Zhou et al., 2016) recommendations regarding postoperative follow-up for TKA patients. Follow-up assessments were conducted at 1 month (T1), 3 months (T2), and 6 months (T3) post-surgery. Before data collection, the researcher explained the study's purpose, significance, and questionnaire completion procedures to the patients in detail. Informed consent was obtained, and valid contact information was retained. Questionnaires were completed independently by the patients. For patients unable to complete the questionnaires independently, the researcher asked each item verbally and accurately recorded their responses. Baseline general information and preoperative WOMAC scores were collected in person by the researcher before surgery. At the 1-month, 3-month, and 6-month postoperative time points, WOMAC and TSK-11 data were collected via telephone follow-up. Several measures were implemented to safeguard data accuracy and quality during telephone follow-ups. First, rapport-building and clear communication of the follow-up plan occurred during hospitalization. Second, appointments were confirmed via phone two days before each scheduled assessment (at 1, 3, and 6 months) to ensure a quiet, conducive environment for the conversation. Finally, a single trained assessor conducted all interviews using a standardized, neutral script without leading questions. To minimize recording errors, the assessor verbally confirmed patients' responses to scale items by repeating their choices. All collected data were independently verified by two researchers before being entered into an electronic database to ensure accuracy.

### 2.4 Statistical analysis

Data processing and analysis were performed using SPSS 27.0 and Mplus 8.3 software. The normality of continuous variables was assessed using skewness and kurtosis coefficients. Normally distributed data were described using mean ± standard deviation, while non-normally distributed data were described using median (interquartile range). Categorical variables were presented as frequency (percentage). Repeated-measures analysis of variance (ANOVA) was employed to examine the temporal trends of joint function and kinesiophobia across the three time points. Pearson correlation analysis was used to explore the relationships between variables. Common method bias was examined via Harman's single-factor test, in which exploratory factor analysis was applied separately to the items of the WOMAC and kinesiophobia scales. To investigate the longitudinal interplay and potential causal links between joint function and kinesiophobia following total knee arthroplasty, a cross-lagged panel model was adopted. This model elucidates dynamic inter-variable relationships through three key pathways: autoregressive effects, cross-lagged effects, and concurrent correlations (Wen, 2017). By comparing the magnitude and significance of the cross-lagged coefficients, we assessed the predominant direction of influence between the two constructs and their temporal evolution, after accounting for the stability of each variable over time. In constructing the cross-lagged model, model fit was comprehensively evaluated using the following indices: the ratio of chi-square to degrees of freedom (χ^2^/*df* ), comparative fit index (CFI), Tucker-Lewis index (TLI), standardized root mean square residual (SRMR), and root mean square error of approximation (RMSEA). A model was considered to have an acceptable fit if it met the following criteria: χ^2^/*df* < 5, CFI > 0.90, TLI > 0.90, SRMR < 0.08, and RMSEA < 0.08. The significance level for all statistical tests was set at α = 0.05.

## 3 Results

### 3.1 General characteristics of patients undergoing total knee arthroplasty

A total of 262 patients who underwent TKA were initially enrolled in the study. During the postoperative longitudinal follow-up, 20 patients were lost to follow-up (T1: 9; T2: 3; T3: 8). Ultimately, 242 patients completed all three surveys, yielding a follow-up completion rate of 92.37%. Specific reasons for attrition included: 10 patients could not be reached via the provided contact information, 3 voluntarily withdrew from the study, 1 dropped out due to death, and 6 underwent contralateral knee arthroplasty during the follow-up period. The age of the 242 patients in this study ranged from 54 to 82 years, with a mean age of 66.29 ± 8.26 years. Other general demographic and clinical characteristics are presented in Table 1.

**Table 1:** Baseline characteristics of patients undergoing total knee arthroplasty (*n* = 242).

**Variable**	**Sample (*n* = 242)**
**Gender [*****n*** **(%)]**	
Male	50 (20.66)
Female	192 (79.34)
**Marital Status [*****n*** **(%)]**	
Married	207 (85.54)
Unmarried/divorced/widowed	35 (14.46)
**Educational level [*****n*** **(%)]**	
Primary school or below	170 (70.25)
Middle school	49 (20.25)
High school or above	23 (9.50)
**Monthly household income [*****n*** **(%)]**	
< ¥3,000	85 (35.12)
¥3,000–5,000	105 (43.39)
> ¥5,000	52 (21.49)
**Place of residence [*****n*** **(%)]**	
Rural	155 (64.05)
Urban	87 (35.95)
**K-L Grade [*****n*** **(%)]**	
Grade 3	137 (56.61)
Grade 4	105 (43.39)
**Comorbidities [*****n*** **(%)]**	
None	79 (32.64)
Present	163 (67.36)
Duration of KOA (years, Median (IQR))	9.5 (3, 10)
Preoperative WOMAC score (points, Mean ± SD)	55.59 ± 5.86
BMI (kg/m^2^, Mean ± SD)	24.98 ± 3.79
Age (years, Mean ± SD)	66.29 ± 8.26

### 3.2 Common method bias test

All data in this study were collected via patient self-report, which may introduce common method bias. To mitigate this, researchers emphasized the anonymity and confidentiality of the questionnaires to all participants during the data collection phase. Additionally, Harman's single-factor test was employed to assess common method bias. The results showed that the variance explained by the first common factor across the three measurement points was 35.46, 24.99, and 35.19%, respectively, all of which were below the critical threshold of 40% (Podsakoff et al., 2003). This indicates that common method bias was not substantial and that subsequent analyses could proceed.

### 3.3 Joint function and kinesiophobia scores in patients after total knee arthroplasty

Repeated-measures analysis of joint function and kinesiophobia revealed significant differences in the total and subscale scores across the three follow-up time points (*P* < 0.001), with an overall trend of continuous improvement over time. Details are presented in Tables 2, 3. When defining a favorable functional outcome as a WOMAC score < 48 (Copsey et al., 2019), the rates of favorable outcomes at T1, T2, and T3 were 93.8, 98.76, and 100%, respectively. Using the therapeutic index for evaluation (Zheng, 2002), the WOMAC score improved from a preoperative value of (55.59 ± 5.86) points to (25.29 ± 6.17) points at 6 months postoperatively. Based on this index, 3 patients (1.24%) were rated as “excellent,” 172 (71.07%) as “good,” 66 (27.27%) as “fair,” and 1 (0.41%) as “poor.” The overall rate of excellent-to-good outcomes was 72.31% (175/242).

**Table 2:** Comparison of joint function outcomes and dimension scores across three time points (points, X ± S).

**Dimension**	**Time**				***F***	***P***
	**Preoperative**	**Post-op 1 month**	**Post-op 3 months**	**Post-op 6 months**		
Pain	9.80 ± 2.54	5.67 ± 2.29	3.66 ± 1.88	2.22 ± 1.45	1,460.620	< 0.001
Stiffness	3.41 ± 1.18	1.79 ± 1.08	1.05 ± 0.66	0.53 ± 0.56	604.162	< 0.001
Physical function	42.35 ± 3.59	29.95 ± 4.57	26.19 ± 4.65	22.54 ± 4.81	3,575.091	< 0.001
WOMAC total score	55.59 ± 5.86	37.42 ± 6.81	30.89 ± 6.37	25.29 ± 6.17	4,888.324	< 0.001

WOMAC, Western Ontario and McMaster Universities Osteoarthritis Index.

**Table 3:** Comparison of kinesiophobia and its dimension scores across three time points (points, X ± S).

**Dimension**	**Time**			***F***	***P***
	**Post-op 1 month**	**Post-op 3 months**	**Post-op 6 months**		
Activity cognition	15.03 ± 3.20	12.96 ± 3.11	10.97 ± 3.00	763.159	< 0.001
Activity behavior	8.71 ± 1.61	7.35 ± 1.53	6.33 ± 1.47	240.000	< 0.001
Activity attitude	4.34 ± 0.94	3.57 ± 0.95	2.89 ± 0.83	481.763	< 0.001
TSK-11 total score	28.08 ± 5.33	23.91 ± 5.10	20.24 ± 4.86	2,062.816	< 0.001

TSK-11, Tampa scale for Kinesiophobia-11.

### 3.4 Correlation between joint function outcomes and kinesiophobia in patients after total knee arthroplasty

Pearson correlation analysis in this study revealed significant negative correlations between kinesiophobia and WOMAC scores at all three time points (T1–T3). The correlation coefficients were 0.821, 0.865, and 0.827, respectively. This indicates that poorer joint function outcomes are significantly associated with higher levels of kinesiophobia. The observed correlations meet the prerequisites for conducting cross-lagged panel analysis. Detailed results are presented in Table 4.

**Table 4:** Correlation analysis between joint function outcomes and kinesiophobia across three time points.

**Variable**	**WOMAC T1**	**WOMAC T2**	**WOMAC T3**	**Kinesiophobia T1**	**Kinesiophobia T2**	**Kinesiophobia T3**
WOMAC T1	1	0.901^**^	0.813^**^	0.821^**^	0.828^**^	0.818^**^
WOMAC T2		1	0.901^**^	0.860^**^	0.865^**^	0.866^**^
WOMAC T3			1	0.804^**^	0.809^**^	0.827^**^
Kinesiophobia T1				1	0.939^**^	0.921^**^
Kinesiophobia T2					1	0.937^**^
Kinesiophobia T3						1

^**^P < 0.01.

WOMAC, Western Ontario and McMaster Universities Osteoarthritis Index; T1, T2, T3 = 1, 3, and 6 months postoperatively, respectively.

### 3.5 Cross-lagged analysis between joint function outcomes and kinesiophobia

A cross-lagged panel analysis was conducted to examine the relationship between joint function outcomes and kinesiophobia in patients after TKA. The model demonstrated good fit to the data: CFI = 0.984, TLI = 0.971, RMSEA = 0.064, SRMR = 0.022. The specific cross-lagged regression model is illustrated in Table 5. The results revealed significant autoregressive effects for both joint function and kinesiophobia across all three time points, as well as significant cross-lagged relationships between them. The cross-lagged paths indicated that during the early postoperative phase (T1–T2), kinesiophobia was a stronger predictor of subsequent joint function (β = 0.368, *SE* = 0.092, *P* < 0.001) than joint function was of subsequent kinesiophobia (β = 0.175, *SE* = 0.039, *P* < 0.001). Conversely, during the middle-to-late phase (T2–T3), joint function became a stronger predictor of subsequent kinesiophobia (β = 0.218, *SE* = 0.049, *P* < 0.001) than kinesiophobia was of subsequent joint function (β = 0.114, *SE* = 0.052, *P* = 0.028). These findings suggest that the predominant direction of influence between these two constructs evolves during the rehabilitation process. The relationship shifts from a pattern dominated by psychological factors (kinesiophobia) in the early phase to one increasingly dominated by functional status in the middle-to-late phase, indicating a dynamic, time-sequential interaction.

**Table 5:** Standardized path coefficients of the cross-lagged model between joint function outcomes and kinesiophobia across three time points.

**Item**	**β**	**SE**	**95% CI**	***P*-value**
**Autoregressive paths**				
WOMAC T1 → WOMAC T2	0.559	0.098	(0.407, 0.791)	< 0.001
WOMAC T2 → WOMAC T3	0.803	0.052	(0.717, 0.905)	< 0.001
Kinesiophobia T1 → Kinesiophobia T2	0.796	0.038	(0.722, 0.869)	< 0.001
Kinesiophobia T2 → Kinesiophobia T3	0.749	0.047	(0.658, 0.840)	< 0.001
**Cross-lagged paths**				
WOMAC T1 → Kinesiophobia T2	0.175	0.039	(0.098, 0.252)	< 0.001
WOMAC T2 → Kinesiophobia T3	0.218	0.049	(0.121, 0.314)	< 0.001
Kinesiophobia T1 → WOMAC T2	0.368	0.092	(0.188, 0.548)	< 0.001
Kinesiophobia T2 → WOMAC T3	0.114	0.052	(0.012, 0.216)	0.028
**Correlations**				
WOMAC T1 ↔ Kinesiophobia T1	0.522	0.051	(0.422, 0.623)	< 0.001
WOMAC T2 ↔ Kinesiophobia T2	0.193	0.064	(0.067, 0.319)	0.003
WOMAC T3 ↔ Kinesiophobia T3	0.175	0.057	(0.064, 0.283)	0.002

β, standardized path coefficient; SE, standard error; CI, confidence interval; WOMAC, Western Ontario and McMaster Universities Osteoarthritis Index; T1, T2, T3 = 1, 3, and 6 months postoperatively, respectively.

## 4 Discussion

### 4.1 Joint function outcomes following total knee arthroplasty improve over time

The results of this study indicate that scores across all WOMAC domains, as well as the total score, showed a progressive decline over time in TKA patients, reflecting gradual improvements in pain, stiffness, and physical function. These findings align with those reported by Lu et al. (2025). The rates of good-to-excellent functional outcomes at the three follow-up time points were 93.80, 98.76, and 100%, respectively, demonstrating that TKA effectively improves joint function, restoring most patients to a mild-to-moderate functional level, consistent with the conclusions of Wright et al. (2010). However, evaluation based on the therapeutic index revealed that the rate of good-to-excellent outcomes at 6 months postoperatively was 72.31%, suggesting that approximately 27.69% of patients did not achieve the desired level of functional improvement. This discrepancy may be related to the severity of preoperative joint damage and individual variations in rehabilitation progress. In this study, patients had a long disease duration with recurrent symptoms, and a substantial proportion (43.39%) presented with Kellgren-Lawrence grade 4 osteoarthritis. Existing research indicates that although patients with poorer preoperative function often exhibit greater absolute improvement, their relative recovery level may be lower compared to patients with better preserved preoperative function due to their lower baseline (Jo et al., 2018). Recent years have seen increasing international focus on delineating functional recovery trajectories. Several prospective longitudinal studies using latent class models have identified 2–3 distinct recovery trajectories among TKA patients, highlighting the heterogeneity in rehabilitation processes (Riddle et al., 2022; Dumenci et al., 2019; Hamilton et al., 2021). Therefore, clinical practitioners should prioritize postoperative follow-up. By leveraging follow-up management systems to collect data on joint function and quality of life, future research can investigate multi-trajectory recovery patterns. This knowledge is crucial for constructing and refining precise, personalized rehabilitation protocols for TKA patients, ultimately aiming to achieve higher-quality postoperative recovery.

### 4.2 Kinesiophobia in TKA patients decreases over time

According to the Fear-Avoidance Model, kinesiophobia can directly lead to adaptive avoidance behaviors in patients, such as limping and inactivity. Prolonged avoidance, in turn, can trigger a series of adverse outcomes, including disuse syndrome, depression, and functional disability (Filardo et al., 2017). These results demonstrate a progressive postoperative decrease in kinesiophobia among TKA patients, aligning with the evidence from a prior prospective cohort study by Yan et al. (2023) involving a similar population. This trend is likely associated with postoperative pain reduction, improved joint function, and alleviation of physical discomfort. Research confirms that pain symptoms and joint function in TKA patients show progressive improvement over the recovery period, reaching optimal levels within 1 year post-surgery (Naylor et al., 2009). However, Zhang et al. (2024) found heterogeneity in kinesiophobia levels among TKA patients at 6 months postoperatively. Older patients had more comorbidities, lower social support, lower self-efficacy, and a tendency toward pain catastrophizing, and exhibited more severe and persistent kinesiophobia. Notably, pain intensity was not an independent predictor of high kinesiophobia. These findings collectively suggest that kinesiophobia should not be considered merely a derivative of pain. It must be addressed as an independent intervention target. Future efforts should focus on the early identification of patients at high risk for kinesiophobia. Several evidence-based strategies can be applied across the continuum of care for kinesiophobia. As a foundational preventive approach, activity exposure, based on exposure therapy principles, has established efficacy (Blanchard et al., 2022). For therapeutic intervention, Pain Neuroscience Education (PNE), a cognitive-behavioral approach, is effective in modifying maladaptive pain cognitions (Larsen et al., 2024). Additionally, Acceptance and Commitment Therapy (ACT) promotes psychological flexibility by encouraging acceptance of discomfort and engagement in value-directed actions, which also reduces kinesiophobia (Farris and Kibbey, 2022). Therefore, for patients exhibiting severe akinesia in the immediate postoperative period, PNE may be promptly introduced to facilitate cognitive restructuring, alongside initiating a personalized graded motor exposure programme. For those experiencing persistent akinesia during recovery, enhanced ACT training may be employed to improve psychological flexibility.

### 4.3 Cross-lagged analysis of joint function and kinesiophobia after total knee arthroplasty

The cross-lagged analysis revealed a significant and dynamic bidirectional relationship between joint function and kinesiophobia in patients following TKA. The shift in the predictive strength between these two variables from the early (T1–T2) to the middle-to-late (T2–T3) phase indicates a staged change in the dominant influencing factors during the rehabilitation process. In the early rehabilitation phase, kinesiophobia demonstrated a stronger predictive power over subsequent joint function, suggesting that it is a core factor constraining functional recovery during the initial postoperative period. This aligns with the findings of Fan et al. (2025). Analysis indicates that within the first 8 weeks post-TKA, the knee remains in the acute physiological healing phase following surgical trauma. Tissue edema, inflammatory responses, and pain constitute normal protective signals from the body. At this stage, patients typically maintain vigilance toward pain and spontaneously reduce activity, which reflects an adaptive response that helps protect the surgical site and prevent secondary injury. However, in some patients, heightened sensitivity to pain, insufficient knowledge of rehabilitation protocols, or the influence of catastrophic thinking may amplify or prolong this transient physiological protective response. This can foster excessive apprehension regarding the safety of rehabilitation exercises and the stability of the prosthesis, leading to an overestimation of activity risks and the development of persistent avoidance behaviors, ultimately hindering the restoration of joint function (Zhou et al., 2023). Consequently, during this early stage, the focus of rehabilitation should be on psychologically helping patients establish correct cognitions about movement, rather than solely emphasizing exercise volume. Healthcare professionals should, on the basis of effective analgesia, conduct early psychological screening using standardized assessment tools such as kinesiophobia and pain catastrophizing scales to identify patients' specific fear content (Luna et al., 2017; Kazarian et al., 2021). Personalized interventions, including education, psychological counseling, and cognitive-behavioral strategies, should then be implemented based on this assessment (Liu et al., 2024; Meyer et al., 2023). Later, during bedside rehabilitation, therapists can use graded exposure therapy, allowing patients to experience successful activities firsthand that confirm safety within specific limits (Blanchard et al., 2022). After each activity, patients should be guided to compare their catastrophic expectations with the actual outcomes. As avoidance behaviors decrease, control over activities can be gradually transferred back to the patient, preparing them for home-based rehabilitation.

As rehabilitation progresses into the middle and late phases, the predictive effect of joint function on subsequent kinesiophobia becomes more prominent, a finding consistent with studies by Filardo et al. (2016). Analysis indicates that over time, sustained physiological improvements promote “joint amnesia”—a state in which patients gradually cease to perceive the artificial joint during routine activities consciously. This represents the ideal functional outcome targeted by total knee arthroplasty (Li et al., 2024). As daily movements become increasingly automatic, patients receive ongoing positive reinforcement from successful rehabilitation experiences. This process establishes a virtuous cycle wherein functional gains and psychological adaptation reciprocally reinforce each other. This positive feedback enhances self-efficacy, which in turn increases the frequency and intensity of rehabilitation exercises, making functional status itself a key factor in alleviating kinesiophobia (Candiri et al., 2023). Based on this finding, clinicians should establish a goal-oriented continuum of rehabilitation that spans the preoperative to the postoperative phases. By facilitating progressive, perceptible improvements, this approach enhances patients' self-efficacy and treatment adherence, thereby promoting synergistic functional and psychological recovery. This continuum begins in the preoperative period, where prehabilitation and structured preoperative education serve as foundational components. Prehabilitation systematically optimizes physical fitness, nutritional status, and psychological readiness, thereby increasing physiological and psychological reserve; evidence supports its role in improving early postoperative functional outcomes (Konnyu et al., 2023). Concurrently, preoperative education prepares patients psychologically by managing expectations, reducing anxiety, and mitigating catastrophic thinking, setting the stage for adaptive recovery (Ho et al., 2022; Moyer et al., 2017). Building on this foundation, postoperative rehabilitation should be dynamically adapted according to phased, task-specific principles. In the early phase (e.g., within the first month), rehabilitation should emphasize restoring joint range of motion and improving lower limb muscle strength through modalities such as stretching and mobility exercises, while simultaneously addressing kinesiophobia (Chen et al., 2024). As patients transition into the stable recovery phase (e.g., around 3 months postoperatively), training objectives can advance to enhancing balance, maximizing muscle strength, and refining motor control (Chen et al., 2024). At this stage, activities such as simplified Tai Chi may be incorporated (Chen et al., 2024). This progression through goal-oriented, task-based activities allows patients to experience continuous positive reinforcement through observable gains, which further strengthens self-efficacy and adherence. Ultimately, this integrated approach fosters the synergistic development of joint function and psychological wellbeing.

## 5 Conclusion

Using a cross-lagged panel model, this study longitudinally revealed a bidirectional and time-ordered dynamic relationship between kinesiophobia and joint function after TKA. It identified the dominant factors at different rehabilitation stages, suggesting a focus on psychological interventions in the early phase and enhanced rehabilitation exercises in the mid-to-late phases. These findings provide new empirical evidence for understanding the psychophysiological interactions during postoperative recovery.

However, this study has several limitations. First, as an observational study with a sample drawn from only two hospitals and without prospective registration of the study protocol on a public platform, the generalizability of the results may be limited, and there is a risk of selective reporting. Second, although all surgeries employed cemented fixation and conventional instrumentation, excluding navigation and robotic-assisted techniques, specific prosthesis subtypes and surgical approaches were not restricted. Therefore, the conclusions are more applicable to patients undergoing mainstream, conventional surgical protocols. Finally, the follow-up period of 6 months, while covering the major rehabilitation phase, did not allow observation of the long-term steady-state relationship between joint function and kinesiophobia. A longer follow-up is crucial to investigate whether early kinesiophobia exerts a sustained influence on long-term “joint amnesia” and patient satisfaction. Based on these limitations, future research could advance in the following directions: Conduct prospective, registered, multicenter, long-term cohort studies to validate the present model and explore long-term trajectories; Systematically collect and analyze surgical details in the study design to build more precise predictive models; Using the “stage-specific” findings from this study as a foundation, design and validate the efficacy of a “stage-targeted” combined intervention protocol based on this dynamic relational theory.

## Data Availability

The original contributions presented in the study are included in the article/supplementary material, further inquiries can be directed to the corresponding author.
